# Increased Risk of Wheeze and Decreased Lung Function after Respiratory Syncytial Virus Infection

**DOI:** 10.1371/journal.pone.0087162

**Published:** 2014-01-31

**Authors:** Kim Zomer-Kooijker, Cornelis K. van der Ent, Marieke J. J. Ermers, Cuno S. P. M. Uiterwaal, Maroeska M. Rovers, Louis J. Bont, on behalf of the RSV Corticosteroid Study Group

**Affiliations:** 1 Department of Paediatric Pulmonology and Allergology, Wilhelmina Children’s Hospital, University Medical Centre Utrecht, Utrecht, The Netherlands; 2 Department of Psychiatry, University Medical Centre Utrecht, Utrecht, The Netherlands; 3 Julius Centre for Health Sciences and Primary Care, University Medical Centre Utrecht, Utrecht, The Netherlands; 4 Department of Epidemiology, Biostatistics & HTA and operating rooms, Radboud University Medical Centre Nijmegen, Nijmegen, The Netherlands; 5 Department of Paediatric Immunology and Infectious Diseases, Wilhelmina Children’s Hospital, University Medical Centre Utrecht, Utrecht, The Netherlands; University of Iowa, United States of America

## Abstract

**Background:**

A relationship between hospitalization for respiratory syncytial virus (RSV) bronchiolitis and asthma development has been suggested in case-control studies.

**Objective:**

The aim of this study was to assess the risk of current wheeze, asthma, and lung function at school age in infants previously hospitalized for RSV bronchiolitis compared to non-hospitalized children.

**Methods:**

For this study, data from a prospective birth cohort of unselected, term-born infants (n = 553), of whom 4 (0.7%) were hospitalized for RSV bronchiolitis, and a prospective patient cohort of 155 term infants hospitalized for RSV bronchiolitis were used. Respiratory outcomes at age 6 in children hospitalized for RSV bronchiolitis were compared to non-hospitalized children.

**Results:**

The risk of current wheeze was higher in hospitalized patients (n = 159) compared to non-hospitalized children (n = 549) (adjusted odds ratio (OR) 3.2 (95% CI 1.2–8.1). Similarly, the risk of current asthma, defined as a doctor’s diagnosis of asthma plus current symptoms or medication use, was higher in hospitalized patients (adjusted OR 3.1 (95% CI 1.3–7.5). Compared to non-hospitalized children, RSV bronchiolitis hospitalization was associated with lower lung function (mean difference FEV1% predicted −6.8 l (95% CI (−10.2 to −3.4).

**Conclusions and Clinical Relevance:**

This is the first study showing that hospitalization for RSV bronchiolitis during infancy is associated with increased risk of wheezing, current asthma, and impaired lung function as compared to an unselected birth cohort at age 6.

## Introduction

Respiratory syncytial virus (RSV) infection is a common cause of severe bronchiolitis in infants. The annual global incidence of RSV infection in children younger that 5 years, has been estimated at 34 million per year, with at least 10% episodes representing severe infections that require hospitalization [1]. Over the last 20 years, epidemiologic studies have shown an association between RSV bronchiolitis and the subsequent development of wheeze and asthma up to school age [2]. Most studies had a case-control design [3]–[7], which bears the challenge of control selection. In order to prevent selection bias, sampling of controls needs to be independent of the determinant studied [8]. The ALSPAC study retrospectively analyzed the relationship between a discharge code of hospitalization for RSV bronchiolitis and long-term airway disease up to age 7 years within in the study registry [9]. In that study there was an excess of subjects with missing data among RSV cases. A recent meta-analysis concluded that study quality of follow-up studies after RSV bronchiolitis was generally poor [10]. For that reason Stein and Martinez argued that the association between RSV hospitalization during infancy and asthma at school has still not been established [11]. To our knowledge, no study has prospectively compared wheezing and lung function at school age between term hospitalized RSV bronchiolitis patients and a large birth cohort of healthy term children. We performed a prospective cohort study of healthy infants, including a group of well characterized, hospitalized RSV infants with bronchiolitis [12]. Our aim was to determine whether the risk of wheeze and asthma at age 6 is related to RSV bronchiolitis hospitalization during infancy.

## Materials and Methods

### Participants

The WHISTLER project is an ongoing population based, prospective birth cohort on determinants of wheezing illnesses in children, which started in December 2001. Participants are healthy, term-born infants born in Leidsche Rijn, a residential area near the city of Utrecht. Infants are enrolled at the age of 2 weeks, and are prospectively followed with (1) a diary for respiratory symptoms in the first year of life, and with (2) study visits including lung function at age 5 and 8 [12]. For each participant, data from the most recent study visit was used (either from the visit at age 5 or age 8), resulting in 553 children of similar age for analysis. Children that were hospitalized during infancy for a proven RSV bronchiolitis (positive immunofluorescence test or PCR) were identified. This birth cohort was compared to a cohort of children that had been hospitalized for RSV bronchiolitis. Summarized, between 2004 and 2006, 243 previously healthy infants aged less than 13 months were admitted to the hospital with an RSV infection, as described earlier in detail [13]. Patients with a proven RSV bronchiolitis by a positive immunofluorescence test for RSV in epithelial cells from nasopharyngeal aspirates were randomly assigned to receive either high dose extra fine HFA beclomethasone dipropionate or placebo. At age 6, no differences in lung function or presence of respiratory symptoms were found between groups, and therefore we combined both groups for the current report. All children were invited to participate in the follow-up study visit at 6 years of age, including a questionnaire, and lung function assessment. From April 2010 to November 2011, 185 (76%) children attended this study visit (Figure S1). Only term born infants (155/185) were included, and added to the 4 hospitalized RSV bronchiolitis patients in the Whistler cohort. Respiratory outcomes of hospitalized RSV bronchiolitis patients within the complete cohort (n = 159, hereafter called hospitalized patients), were compared to non-hospitalized children (n = 549). Parents gave written informed consent for study participation.

### Ethics Statement

The medical ethics committee of the University Medical Centre Utrecht approved both studies. Written informed parent consent was obtained from all parents. The study was conducted according to the principles of the Declaration of Helsinki (version 2000) and in accordance with the Dutch Medical Research Involving Human Subjects Act (WMO). Good Clinical Practice (GCP) guidelines were followed.

### Outcomes

#### Questionnaire and definitions

The questionnaire contained standardized questions about atopic diseases from the ISAAC questionnaire [14], medication use (e.g. antihistamines, inhalation steroids and beta-mimetics) in the last 12 months and known risk factors for atopic diseases (i.e. positive family history). All questions were filled in by the parents of the hospitalized patients and non-hospitalized children. Parental allergy was defined as a questionnaire-reported allergy to pollen, house dust mite, pets or food. Smoke exposure was defined as smoking of one of the parents in the last 5 years of life, and data from the first year of life were used to obtain information about smoke exposure in the first year of life to prevent recall bias. A non-western origin was defined as a country of birth in Asia (including Turkey), Africa, Latin America, excluding Indonesia and Japan. In the Netherlands, children regularly visit child healthcare centers for standardized anthropometry. These measurements are recorded in a personal file for every child, kept by parents. Parents were asked to use this file to report these anthropometric measures in the questionnaire. Current wheeze, in both the hospitalized patients and non-hospitalized children, was defined as a positive response to the question “Has your child had wheezing or whistling in the chest in the last 12 months?” Current asthma was defined as a history of doctor’s diagnosis of asthma plus asthma symptoms or medication (beta-mimetics or inhaled corticosteroids) use in the last 12 months.

A doctor’s diagnosis of asthma ever was defined as an ever recorded asthma diagnosis from the general practitioner after a primary care visit (R03 wheezing, R96 asthma), according to the International Classification of Primary Care (ICPC) [15].

#### Lung function

Spirometry was performed using a calibrated spirometer (Zan 100 pulmonary spirometer system (nSpire, USA). Maximal flow-volume curves were measured according to the ATS/ERS standards [20]. The largest forced expiratory volume in 1 second (FEV_1_), forced vital capacity (FVC) and peak expiratory flow (PEF) were selected from three correctly performed manoeuvres. Results were related to Dutch normative values [16]. Expiratory measurements of respiratory system resistance (R_int_) were obtained using MicroRint (Micro Medical Limited, Kent, UK). Mean R_int_ was calculated from at least 5 acceptable interruptions. FeNO was measured in exhaled breath using the NioxMino (NIOX; Aerocrine AB, Solna, Sweden). All children withheld their rescue medication for at least 12 hours beforehand. If the child had suffered from a respiratory tract infection in the last 2 weeks, the test was postponed.

#### Statistical analysis

Logistic regression was used to investigate the effect of RSV bronchiolitis hospitalization on current wheeze and asthma. Hereafter, multivariable analysis was used to adjust for possible confounders influencing both current wheeze or asthma (outcomes) as well as RSV bronchiolitis hospitalization (determinant). Results are presented as odds ratio (OR) with a 95% confidence interval (CI). Linear regression was used to investigate the effect of RSV bronchiolitis hospitalization on lung function. Again, multivariable analyses was used to adjust for possible confounders, and results are presented as crude and adjusted mean differences with a 95% CI. Data were analyzed using PASW Statistics 18 (version 18.0.0, SPSS Inc., 2009, Chicago USA).

#### Confounders

Baseline characteristics between the two cohorts were likely to be different with respect to risk factors for RSV bronchiolitis hospitalization. These risk factors were considered to be potential confounders of the association between RSV hospitalization and current wheeze or asthma. The following confounders were corrected for in our analysis: male gender, breastfeeding, siblings, day care attendance, smoke exposure, birth weight, being born between April and September, year of birth, and educational level of the mother.

## Results

Of the 243 hospitalized RSV bronchiolitis patients that participated in the initial trial, 185 participated in the follow-up study. 155 Previously healthy, term infants were selected for further analyses, of whom 113 (72.9%) agreed to take part in the lung function test (figure S1). Non-participants were more often of non-Caucasian ethnicity compared to participants or the excluded premature born participants (table S1). Participants with a successful lung function measurement were significantly younger compared to participants with unsuccessful lung function measurement (5.8 versus 6.3 years, p<0.001, table S2). Children hospitalized for RSV bronchiolitis had lower birth weight, were more often born between September and March, more often exposed to maternal smoking in pregnancy, less often breastfed, did go to daycare less often, had more often siblings, had lower educated parents and more often Caucasian parents compared to non-hospitalized children (table 1).

**Table 1:** Group characteristics at age 6 for 159 hospitalized RSV bronchiolitis patients [13], and non-hospitalized children [12].

	Hospitalized RSVbronchiolitis patients(n = 159)	Non-hospitalized (n = 549)	p-value
Sex (male)	87/159 (54.7)	265/549 (48.3)	0.153
Median age at follow-up in yrs (IQR)	5.88 (5.67–6.25)	5.33 (5.17–7.75)	0.923
Median age at hospitalisation (months)	2.0 (1.0–4.0)	–	–
Birth weight (g)	3250 (3250–3750)	3557 (3260–3860)	<0.001
Gestational age (weeks)	40.0 (39.0–40.6)	40.0 (39.1–40.9)	0.081
Born September-March	99/159 (62.3)	270/549 (49.2)	0.002
Smoking during pregnancy	27/155 (17.0)	26/548 (4.7)	<0.001
Smoke exposure up to age 6	42/159 (26.4)	126/451 (27.9)	0.721
Breastfed in first 3 months	97/155 (62.6)	3768/515 (71.5)	0.036
Daycare in first 3 months	52/155 (33.5)	227/515 (44.1)	0.020
Pets in first year of life	79/154 (51.3)	274/475 (57.7)	0.166
Siblings	134/155 (86.5)	297/548 (54.2)	<0.001
Maternal atopy	66/154 (42.9)	224/466 (48.1)	0.262
Maternal ethnicity Caucasian*	149/154 (96.8)	386/469 (82.3)	<0.001
Maternal high educational level	53/153 (34.6)	301/470 (64.0)	<0.001
Median height at age 6 (cm, IQR)	117.2 (113.0–126.10	118.2 (114.3–121.8)	0.094
Median weight at age 6 (kg, IQR)	21.0 (19.0–25.0)	21.6 (19.7–24.0)	0.567

Data are numbers (percentages) unless stated otherwise.

* Caucasian = Not born in Africa, Latin America and Asia (Japan and Indonesia excluded) or Turkey.

### Current Wheeze, Asthma and Allergic Diseases

In the univariable logistic regression analysis hospitalized RSV bronchiolitis patients had a 3.2 fold increased odds for wheeze in the last 12 months (table 2). Hospitalized RSV patients more often were diagnosed with asthma at any point in life, and a higher proportion had current asthma defined as a history of doctor’s diagnosis of asthma plus asthma symptoms or medication use in the last 12 months. Hospitalized patients more often used airway medication compared to non-hospitalized children, however this did not reach statistical significance. In multivariable analysis we initially adjusted the risk of current wheeze and asthma for sex and age only, but this did not change our results. We repeated our analysis by adjusting for sex, age, birth weight, season of birth, year of birth, smoke exposure during pregnancy and during life, breastfeeding, daycare, siblings, maternal atopy and maternal ethnicity. This slightly changed the odds for current wheeze and current asthma to 3.2 (95% CI 1.2 to 8.1) and 3.1 (95%CI 1.3 to 7.5) respectively.

**Table 2:** Respiratory morbidity of hospitalized RSV bronchiolitis patients and non-hospitalized children at the age of 6 years.

Respiratory morbidityat age 6	HospitalizedRSVbronchiolitispatients	Non hospitalized	Crude			Adjusted*			Adjusted**		
	(n = 159)	(n = 549)	OR	95% CI	p-value	OR	95% CI	p-value	OR	95% CI	p-value
Respiratory symptoms											
Current wheeze	33/155 (21.3)	42/516 (8.1)	3.0	1.9–5.0	<0.001	3.0	1.9–5.1	<0.001	3.2	1.2–8.1	0.002
Current use of asthmamedication											
Inhaled steroids	11/159 (6.9)	25/549 (6.9)	1.6	0.7–3.2	0.235	1.5	0.7–3.2	0.255	3.4	1.0–11.3	0.292
Betamimetics	17/159 (10.7)	46/549 (8.4)	1.3	0.7–2.4	0.368	1.3	0.7–2.3	0.378	2.1	0.8–5.7	0.109
Asthma											
Doctor’s diagnosisasthma ever	63/158 (39.9)	58/517 (11.2)	5.2	3.5–8.0	<0.001	5.3	3.4–8.0	<0.001	5.8	2.8–11.8	<0.001
Current asthma***	34/159 (21.4)	29/549 (5.3)	4.9	2.9–8.3	<0.001	4.8	2.8–8.2	<0.001	3.1	1.3–7.5	0.010

Data are numbers (percentages) unless stated otherwise;

* Adjusted for sex and age;

** Adjusted for sex, age, birth weight, birth season, smoke exposure during pregnancy and during life, breastfeeding, daycare, siblings, maternal atopy, ethnicity, year of birth, and maternal educational level;

*** Defined as combination of a history of doctor’s diagnosed asthma plus asthma symptoms or medication use in the last 12 months (beta-mimetics or inhaled corticosteroids).

Comparable adjusted odds ratios were found for current wheeze in atopic children (OR 3.7 (95% CI 0.8 to 15.6) and non-atopic children (2.9 (95%CI 0.8 to 10.5), as well as for current asthma in atopic (OR 3.3 (95% CI 0.8 to 13.6) and non-atopic children (2.6 (95%CI 0.8 to 8.8)). Proportions of parent-reported allergic diseases in their children were not significantly different between the hospitalized patients and non-hospitalized children (hay fever 5.7% versus 5.5%, and eczema 34.0% versus 27.5%).

### Lung Function

**Figure 1:**
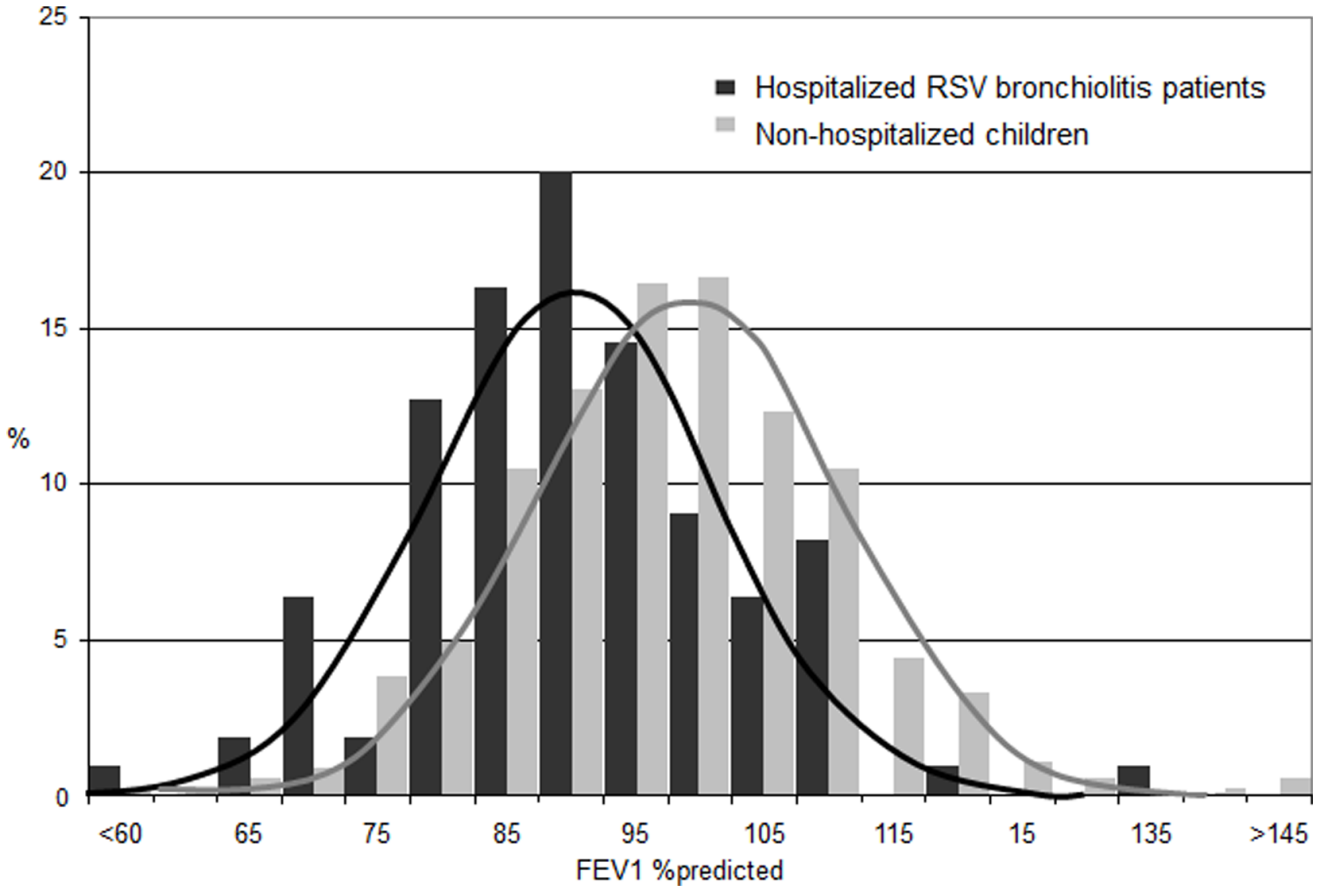
FEV_1_ values presented as % predicted values for hospitalized RSV bronchiolitis patients and non-hospitalized children measured at the age of 6 years. Hospitalized patients had a lower mean FEV_1%_ predicted compared to non hospitalized children (93.3 (SD12.2) versus 100.3% (SD 13.9), mean difference −7.0 (95% CI (−9.7 to −4.2)).

Compared to the non-hospitalized children, lung function was significantly impaired in the hospitalized RSV patients (table 3, figure 1). Hospitalized patients had lower FEV_1_ (mean difference (MD) −6.8 l. %predicted (95% CI −10.2 to −3.4), lower FVC (MD −6.5 l. %predicted) (95% CI −10.4 to −2.7), a lower Tiffeneau index (MD −2.0% (95% CI −3.6 to −0.3), higher FeNO (MD 4.0 ppb (95% CI 1.9 to 6.2) and increased resistance of the respiratory system (MD 15.6 kPa/L/s (95% CI 6.5 to 24.6) compared to the non-hospitalized children.

**Table 3:** Lung functions of hospitalized RSV bronchiolitis patients and non-hospitalized children at the age of 6 years.

	Hospitalized RSVbronchiolitis patients			Non-hospitalized			Crude			Adjusted*		
	(n = 117)			(n = 549)								
		Absolute^#^	% predicted		Absolute^#^	% predicted		Mean difference (95% CI)	p-value		Mean difference(95% CI)	p-value
FEV_1_ (l)		1.24 (0.20)	93.3 (12.19)		1.43 (0.33)	100.27 (13.90)		−6.97 (−9.69 to −4.24)	<0.001		−6.82 (−10.20 to −3.44)	<0.001
FVC (l)		1.35 (0.24)	98.16 (13.81)		1.53 (0.39)	104.31 (15.55)		−6.16 (−9.21 to −3.11)	<0.001		−6.54 (−10.43 to −2.65)	0.001
FEV_1_/FVC (%)		92.32 (8.68)	–		93.68 (5.96)			−1.36 (−2.75 to 0.04)	0.057		−1.97 (−3.61 to −0.33)	0.019
PEF (l/s)		2.86 (0.49)	98.09 (15.19)		3.17 (0.89)	101.36 (21.45)		−3.28 (−7.71 to 1.17)	0.148		−2.93 (−8.59 to 2.73)	0.310
R_int_ (kPa L^−1^ s)		0.76 (0.23)	124.72 (35.69)		0.66 (0.17)	112.73 (31.88)		11.99 (4.54 to 19.43)	0.002		15.59 (6.55 to 24.64)	0.001
FeNO (ppb)^a^		10.85 (9.01)	–		8.12 (5.47)	–		2.73 (1.15 to 4.29)	0.001		4.02 (1.88 to 6.16)	0.001

Values are expressed as % of the predicted values (mean (SD)); Presented mean differences are differences in mean %predicted values;

* Adjusted for sex, age, birth weight, birth season, smoke exposure during pregnancy and during life, breastfeeding, daycare, siblings, maternal atopy, ethnicity, year of birth, and maternal educational level.

## Discussion

In this prospective study we studied the risk of wheeze and asthma after RSV bronchiolitis hospitalization during infancy. We established a 3.2 fold increased risk of current wheeze and a 3.1 fold increased risk of current asthma in hospitalized RSV bronchiolitis patients compared to non-hospitalized children. In hospitalized RSV bronchiolitis patients the mean FEV_1_ percentage predicted was 6.8% lower compared to non-hospitalized children.

To our knowledge, this is the first study comparing prospectively followed hospitalized infants with RSV bronchiolitis to a normal birth cohort, and obtaining valid risk estimates on respiratory morbidity in childhood. Late effects of RSV bronchiolitis requiring hospitalization have previously been studied by others [3]–[5], [9], [17]. A study by Sigurs et al. [3] reported a 2 fold increased risk of “any wheezing” in prospectively followed hospitalized RSV bronchiolitis patients without concomitant chronic disease, whereas Henderson et al. reported a 3.2–6.6 fold increased risk of wheeze in hospitalized RSV patients selected from a large cohort study [9]. Asthma prevalence in the control population was 3% in the Sigurs study, which is much lower than in the general Swedish population [18]. In our non-hospitalized group the prevalence of wheeze and asthma was 8.1% and 5.3% respectively. These numbers are comparable to the prevalence of wheeze and asthma in 6–7 year old children in western surrounding countries [18], indicating that this group reflects the general population of the Netherlands with respect to wheeze and asthma.

Although symptoms may subside with age, lung function has been shown to track over life [19]. Lung functions in the hospitalized RSV patients were impaired compared to the non-hospitalized children, and differences between groups were larger than described in previous reports [4], [20], [21]. Lung function decline could be of vital importance because low lung function in adulthood is one of the strongest predictors of chronic airway obstructive pulmonary disease later on in life [19].

The mechanism underlying long-term consequences of severe RSV bronchiolitis is intriguing. Here, we confirm the results from the Tucson study showing that atopy does not play a major role in the development of wheeze and loss of lung function following RSV infection. Numerous hypotheses explaining the association between RSV bronchiolitis and asthma development have been published, including innate immune mechanisms, adaptive immune mechanisms as well as neurogenic mechanisms [22]. RSV bronchiolitis is associated with a strong local neutrophil response. Production of proteases and radical oxygen species may cause major damage to the airways resulting in abnormal development of lung architecture and function. An alternative hypothesis is based on our previous observations that children with severe RSV bronchiolitis have a stronger local IL-10 response, which may partially be genetically determined [23]–[25]. Increased local IL-10 production is associated with decreased production of type I and type 2 interferons, leaving patients susceptible to respiratory viral infections.

Previous studies as well as ours were not designed to determine whether RSV bronchiolitis causes airway morbidity at school age or reflects a common predisposition, probably abnormal lung function at birth [26]. It is clear that both hypotheses are not mutually exclusive. We have recently shown that RSV infection is causally related to recurrent wheeze in the first year of life in otherwise healthy preterm infants 33–35 weeks gestational age [27]. RSV immunoprophylaxis prevented 60% of all cause wheeze, even after end of therapy. Similarly, intervention studies are required to assess to what extent RSV hospitalization causes asthma at school age, which will prove instrumental in estimating the potential long-term benefit of new preventive and therapeutic interventions against RSV, of which many are currently under development [28].

In this study we assessed the unbiased risk of wheeze and asthma in childhood after RSV bronchiolitis hospitalization. However, some limitations need to be discussed. First, although our study has some advantages over previous retrospective or case-control studies, the optimal study design was not met. In our cohort, we have actively enriched the group of children hospitalized for RSV bronchiolitis. This led to the limitation that we were not able to assess absolute respiratory risks of hospitalization for RSV, but we were able to validly estimate relative respiratory risks. Second, lung function measurement was not successful in 42 patients (27%) in the RSV hospitalization group. Surprisingly, children with a successful lung function were younger compared to those without a successful lung function. As the remaining baseline characteristics (demographics) in these children were similar to patients with successful lung function measurements, we think that the lung functions measured are representative of all RSV patients. Third, we were able to obtain follow up data from 76% of the hospitalized RSV cohort. Non-participants were more often non-Caucasian compared to participants, which may limit the generalizability of our results to the non-Caucasian group. Fourth, residual confounding may have influenced our conclusions, even though we aimed to correct for all known potential confounders. Fifth, cohort effects may have influenced our outcomes. Although asthma prevalence has increased over the last decades, the prevalence of wheeze and asthma in our study is most likely not affected by the slight variation in time periods between our cohorts. Finally, we did not assess post bronchodilator reversibility which is thought to have added value to make an asthma diagnosis in children [29].

In summary, this is the first prospective study comparing previously healthy term infants hospitalized for RSV bronchiolitis with an unselected healthy, term population. We established that hospitalization for RSV bronchiolitis was associated with a 3.2-fold increased risk of wheeze, a 3.1-fold increased risk of asthma and a 6.8% decrease in FEV_1_% predicted at age 6 compared to non-hospitalized children. Intervention studies will have to determine to what extent RSV bronchiolitis is causally related to long-term airway disease.

## Supporting Information

Figure S1 **RSV study population at 6 years follow up.**
(TIF)

Table S1 **Baseline characteristics of participants, non-participants and premature participants that were excluded from analyses.**
(DOC)

Table S2 **General characteristics of the population with and without a successful lung function measurement.**
(DOCX)
