# Supplementary figures and images for: Increased Risk of Wheeze and Decreased Lung Function after Respiratory Syncytial Virus Infection
Source: PLoS One. 2014 Jan 31;9(1):e87162. doi: 10.1371/journal.pone.0087162 (PMC3909049; doi:10.1371/journal.pone.0087162)

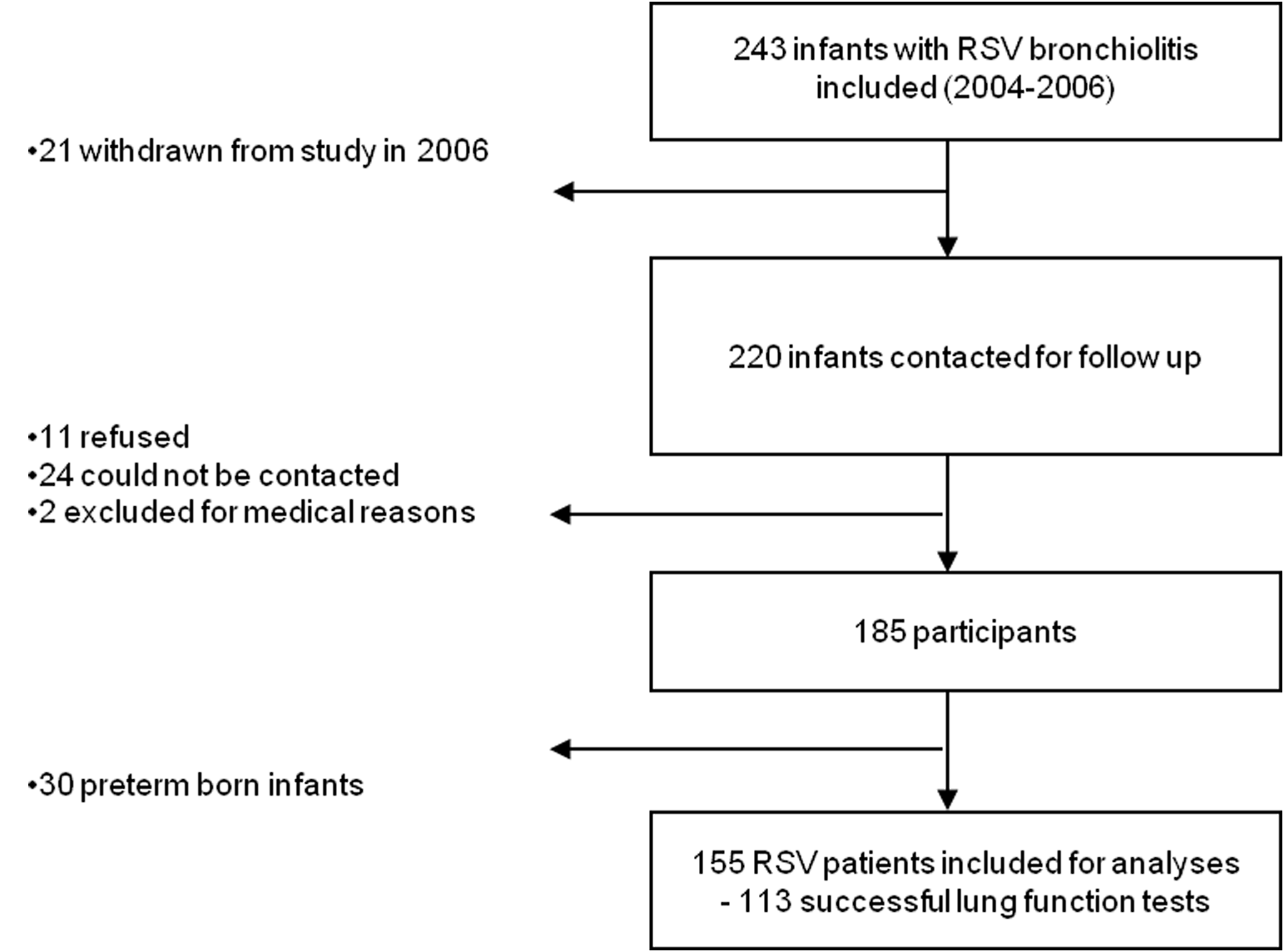

Supplement: Figure S1 — RSV study population at 6 years follow up. (TIF) [file pone.0087162.s001.tif]
