# Supplementary material for: Increased Risk of Wheeze and Decreased Lung Function after Respiratory Syncytial Virus Infection
Source: PLoS One. 2014 Jan 31;9(1):e87162. doi: 10.1371/journal.pone.0087162 (PMC3909049; doi:10.1371/journal.pone.0087162)
Supplement: Table S1 — Baseline characteristics of participants, non-participants and premature participants that were excluded from analyses. (DOC) [file pone.0087162.s002.doc]

**Table S1. Baseline characteristics of participants, non-participants and premature participants that were excluded from analyses. Values are mean (SD) unless stated otherwise**

|  | Participants^a^ | Non-participants^b^ | Premature (non-participant)^c^ | p-value |
| --- | --- | --- | --- | --- |
| Nr of patients | 155 | 58 | 30 |  |
| Sex (male) | 83 (53.5) | 33 (56.9) | 10 (33.3) | 0.089 |
| Median age at follow-up in yrs (IQR) | 5.9 (5.7-6.3) | - | 5.9 (5.8-6.3) | 0.497 |
| Birth weight (g) | 3250 (3250-3500) | 3250 (2750-3500) | 2250 (1750-2750) | <0.001 |
| Maternal atopy | 68/155 (43.9) | 18/37 (48.6) | 10/30 (33.3) | 0.435 |
| Maternal ethnicity Caucasian* | 144/155 (92.9) | 28/58 (48.3) | 29/30 (96.7) | <0.001 |
| Maternal high educational level | 50/150 (33.3) | 9/36 (25.0) | 10/29 (34.5) | 0.303 |

a: patients that attended the study visit for lung function or filled in the questionnaire

b: patients that were lost to follow up, or refused to participate

c: Participants which were excluded from analyses as they were prematurely born
