# Supplementary material for: Increased Risk of Wheeze and Decreased Lung Function after Respiratory Syncytial Virus Infection
Source: PLoS One. 2014 Jan 31;9(1):e87162. doi: 10.1371/journal.pone.0087162 (PMC3909049; doi:10.1371/journal.pone.0087162)
Supplement: Table S2 — General characteristics of the population with and without a successful lung function measurement. (DOCX) [file pone.0087162.s003.docx]

Table S2: General characteristics of the population with and without a successful lung function measurement.

|  | Group with successful LF measurement | Group without successful LF measurement | p-value |
| --- | --- | --- | --- |
| Nr of patients | 113 | 42 |  |
| Sex (male) | 60 (53.1) | 23 (54.8) | 0.853 |
| Median age at follow-up in yrs (IQR) | 5.83 (5.67-6.08) | 6.25 (6.00-6.67) | **<0.001** |
| Gestational age (weeks) | 40.1 (39.1-40.7) | 39.4 (38.3-40.4) | 0.292 |
| Birth weight (g) | 3250 (3250-3750) | 3250 (3250-3750) | 0.759 |
| Maternal atopy | 46/112 (41.1) | 18/39 (46.2) | 0.579 |
| Maternal ethnicity Caucasian* | 110/112 (98.2) | 36/39 (92.3) | 0.109 |
| Maternal high educational level | 40/111 (53.2) | 25/39 (64.1) | 0.458 |

Data are numbers (percentages) unless stated otherwise

*Caucasian = Not born in Africa, Latin America and Asia (Japan and Indonesia excluded) or Turkey.
